# A Case of Nephritis, Terminating in Abscess of the Kidney; Matter Discharged Externally; Recovery

**Published:** 1853-07

**Authors:** L. P. Yandell

**Affiliations:** Professor of Physiology and Pathological Anatomy in the University of Louisville


Art. II.—*A Case of Nephritis, terminating in Abscess of the Kidney; matter discharged externally; recovery.* By L. P. Yandell, M. D., Professor of Physiology and Pathological Anatomy in the University of Louisville.

Mrs. S. J. —., aged 44 years, of good constitution, the mother of twelve children, was attacked on the 4th of September, 1851, with pain in the lumbar region, nausea, and other symptoms of calculus in the kidney. In previous years she had suffered much with similar symptoms, of which she was always relieved after the passage of urinary calculi. The first attack occurred in June, 1843, when six months gone with her eleventh ch[illegible], and from her sufferings she supposed she was about to miscarry, but the discharge of a calculus afforded relief in two or three hours. A week afterwards, another calculus, as large as a pea, was voided, the passage of which along the ureter excited great pain. When nursing her last child, three years subsequently, she suffered much with irritation of the kidneys, induced by the presence of calculi. These, on examination, were found to consist of lithic acid mingled with a large amount of mucus. A visit to Grayson Springs, in April, 1847, improved her health and she had no recurrence of the disorder until the spring of 1848, some months after she had ceased to nurse her child. The attacks in May of that year, while she was on a visit to New York, were frequent and severe, her general health suffering much from the continued renal irritation. On the first of the ensuing August, a calculus was discharged after several hours of intense pain, and from that time she remained free from her complaint up to the date of the last attack. This was preceded for several months by symptoms of gastric disorder; her appetite had been poor and her digestion bad. For several weeks after the first violent symptoms had passed away, she continued uncomfortable, with imperfect digestion, pain and irritation in the region of the left kidney, headache, slight fever, and disturbed sleep. By the use of opiates she finally seemed to obtain relief from these symptoms, and at the beginning of October was considered convalescent.

Oct. 14th. A pain was felt to day in the right side, in the region of the kidney, such as that with which she had suffered so severely in the left, and on the 18th she was seized with a severe, protracted chill. This was followed by others on succeeding days, two, three, and sometimes four occurring in the course of four and twenty hours. She lost flesh rapidly; her complexion, which had been good at the commencement of her illness, became sallow, her skin cool, and her muscular system flaccid. My friends, Profs. Miller and Gross, who had been requested to see my patient, advised the use of iron and bitter tonics. About this time a tumor was detected in her right side, which struck me at once as being connected with the right kidney. After a careful examination, Profs. Drake, Miller and Gross, and Dr. D. W. Yandell formed the same diagnosis of the case. The tumor, which was deep seated and fixed, appeared to be as large as a man's fist, firm, and not painful, except on pressure or the patient's attempting to lie on her left side. The tonic course was kept up for many weeks, but although aided by brandy and a generous diet, she continued to lose flesh; the tumor increased slowly in size and was more painful when she lay on her left side; she had frequent, almost daily, chills, followed by fever; her urine was turbid with mucus, in which the microscope detected crystals of the triple phosphate. On the 23rd of December, a considerable quantity of pus was voided with the urine, and she seemed somewhat relieved by the discharge. Pus corpuscles were detected in the urine for several days. On the 30th of December she made the following memorandum respecting her case:

"To-day I have less acute pain in the upper part of the bladder or right ureter than I had yesterday, and less soreness of my side; yet, upon the whole, I have not felt so well. I am extremely weak; I do not feel that I am stronger than I was a month ago, though my appetite is better. I have a great sense of sinking when I first wake in the morning and until I take some nourishment. The urine, when first discharged, looks clear and natural, but on standing is found to contain a considerable quantity of mucus, with some pus and crystals of the triple phosphate, which under the microscope show beautifully. My bowels are in a perfectly healthy condition, and have been all the time of my sickness, except when I have taken opium. I rest well at night, though my sleep is heavy and not as refreshing as when I was in health. I generally feel much better in the morning than at any other time. I take my breakfast in bed, which consists of milk with crackers, plain corn bread, or buckwheat cakes, and occasionally a little meat. As soon as I rise to dress, though I exert myself as little as possible, I begin to feel faint, with such a sense of sinking that I am often obliged to lie down. And now begins the feeling of general discomfort which clings to me through the day. I am not often in much pain, but in such a state that it requires the greatest effort of the will to change my position. During the morning I drink several glasses of ale. My dinner consists of one kind of meat, and one or two vegetables, with jelly and fruit. My appetite is wayward, and I soon become tired of the same article of diet. I often, during the day, obtain a comfortable position by elevating my feet, sometimes higher than my head. I lie most comfortably with my feet drawn up in bed; in fact I cannot lie with them extended long at a time. I am entirely unable to lie on my left side, owing to the pain it produces in the right kidney and throughout the abdomen. On retiring I generally find it necessary to have a warm iron applied to my feet, which are very cold at that time."

After the discovery of pus in her urine, we began to entertain hopes of her recovery, supposing that the abscess might discharge itself through the ureter and bladder. Her appearance improved, and shortly after she wrote in her diary—" I felt more comfortable throughout the day, and went to bed with more healthful feelings last night, than I have done since I became so ill."

Jan. 10th, she wrote: " Rather comfortable throughout the day; but I gain no strength, and my side is sore and painful."

18th. " The top of my hip, the hip joint and leg down to the knee, were so painful last night that I had to take laudanum. A very sharp, sudden pain across the small of my back this morning. I am daily growing weaker; have less and less ability to move about; my lower extremities, back and loins seem to be somewhat paralysed."

On the 15th she rode out in a carriage, but fancied that the jolting motion increased the pain in the hip and side; the next day, however, she repeated it, and thought she was improved by the ride.

18th. She wrote: "Had a bad night until I took laudanum. My side and hip are excessively painful. I am beginning to feel apprehensive of an outward abscess, my side is so tender to the touch."

A few days later she made the following note of her case: 21st. "Rather an uncomfortable night; more unwell than I have been for weeks; severe pain in my side all day. About 1 o'clock I passed several small calculi, in all forming a mass as large as a pea, black when first voided, but gradually assuming a brown color."

From the extreme tenderness of the tumor and other indications present, it was plain that the abscess which had formed was pointing externally. A blister was applied over the most sensitive part, which drew well and afforded some relief; but in a few days her sufferings were again intense. She wrote on the 24th—

"A most restless and uncomfortable night; my side, and back, and hip could hardly be worse; had to take laudanum."

And again a few days after:

28th. "Had a wretched night; inhaled chloroform almost constantly; feverish, and drank a great deal of water; not a moment's rest all this day."

On consultation with my medical friends, it was decided to attempt to evacuate the contents of the tumor by an operation, and accordingly, on the 28th, Prof. Gross, in presence of Prof. Miller, Dr. Clapp, and Dr. D. W. Yandell, introduced an exploring needle, preparatory to making a larger incision. We were much pained at seeing blood, instead of pus, escape, but it was concluded not to repeat the puncture at that time. Five days after the operation, she wrote:

Feb. 2nd. "My side was so painful that I had to take laudanum, and then a very large pill of opium, last night. I am too feeble to sit up; dull and uncomfortable all the time from taking opium, though it is the only thing that procures rest for me at night. Any change of posture for two days past has produced excruciating pain. Still my appetite is very good; my digestion excellent; and I am free from nausea."

The secretion of urine at this time was copious, owing in a great measure, no doubt, to the freedom with which she indulged in lemonade and effervescing drinks.

6th. Prof. Gross, at my request, punctured the tumor again to-day, and this time with success, more than a pint of pus being discharged, and with signal relief to the patient, who immediately afterwards was able to lie on her left side. A few days previous to the operation she was unable to lie on either side. She passed a very comfortable night, but next day, the discharge having ceased, her side became nearly as painful as ever. The day following the matter made its way spontaneously through the orifice, which had closed temporarily, and afforded perfect relief. She wrote on the 10th:

"Slept well without an opiate; drank but twice during the night; comfortable this morning; side discharging freely."

On the 15th she made the following memorandum;

"I can lie a little on my right side, the swelling and soreness have so diminished; can sit up a minute, and lift my leg a little; my side continues to discharge freely."

On the 20th the abscess was not running, and the patient was first chilly, and then feverish and uncomfortable. She soon became sensible of the dependence of her comfort upon the continuance of the discharge, and was in the habit, when it ceased, of removing the scab from the orifice, which generally renewed it.

25th. Had a slight chill, followed by a good deal of reaction; drank ice water and bathed her feet and hands, which afforded much relief. Abscess not discharging.

26th. Slept well; feels comfortable; fine appetite; sat up several minutes to have her hair dressed. Abscess discharging freely.

March 1st. Rested pretty well; side still runs; no chilliness; very well all day.

4th. Restless, bad night, after 1 o'clock; restless and uncomfortable to-day; soda water constantly. Abscess not discharging.

Thus the patient continued to alternate from day to day, as the matter accumulated or was discharged. On the 15th she wrote:

"Rested well last night, and feel well this morning; side discharging very little; soreness in it diminishing."

About this time she began to be troubled with pain after micturition, and other symptoms which suggested that a calculus had probably passed into the bladder. I sounded for one ten days after, but failed to discover it.

April 2nd. She wrote thus: "I think I shall not again entertain much hope of recovery. I may live a good while as I am, but every thing seems to indicate at present that this is a permanent abscess. It discharges almost constantly, but the discharge has ceased to afford the relief it did at first. I suffer now as much from fever and nausea when it is discharging, as I did for some time when it was not. I gain strength very slowly."

On the 11th—

"Rested well; slight night sweats for a week past; nausea all night. Ate some fat bacon at breakfast this morning, for the first time in a fortnight, and relished it."

Two days after she wrote—

"Rested well, but feel sick this morning, and chilly; the abscess did not discharge yesterday; last night I had the scab removed from the orifice, but no matter has appeared yet. All my symptoms indicate a not very remote termination of my disease—pain in voiding urine, and constant inclination that way; severe occasional pain in the pelvis; nausea; night sweats."

15th. She was carried out of her room and sat up in an arm-chair two or three hours.

23rd. Rested well, and feels pretty well this morning; abscess discharging very little.

27th. After suffering a great deal for two or three days from the presence of a calculus in the bladder, she passed one to day, of a triangular form, as large as a filbert.

29th. Has rested comfortably since the passage of the calculus. Gains strength daily.

May 1st. Side not discharging; bad night; nausea. The same next day; some relief from the nausea by creosote, but still much indisposed; hip very sore.

6th. Rested well; feels better; abscess discharging freely.

9th. Rested well; and although side not discharging, feels quite comfortable.

From this time the abscess discharged very little, but the patient continued to improve daily. She began to move about the house with assistance, but for several months was unable to bear any weight on her right leg, which had become contracted at the knee. Gradually the soreness about the hip and the contraction of the leg wore away, and by the first of November she was able to walk without crutches. She gained flesh, strength, and spirits rapidly by a journey to the South, and now weighs more than she has done in ten years. She still occasionally passes calculous matter, and a month ago was affected for a few hours with severe pain in the left ureter, from the presence of a calculus, but a little laudanum and sulphuric ether afforded her prompt relief.

I think there can be no doubt as to the pathology of this case, which, altogether, is a remarkable one. The disease was nephritis, occasioned by a calculus, resulting in abscess of the kidney. The matter burrowed considerably in its passage towards the surface, and was discharged at a point below the crest of the ilium. It was destitute of odor after the first discharge, and appeared to me well-conditioned No urine or calculous matter ever escaped with it at any time. In the history of the case I have not entered into details of treatment, for the reason that little was attempted in that way, beyond palliating symptoms and supporting the strength of the patient. It was a case in which medicine, apart from this, promised but little.

Of the various outward directions that the pus in renal abscess may take, the one which it pursued in this case is the most common, according to writers on pathological anatomy. Craigie describes the process which takes place in such cases, as the following: "The concretion giving rise to ulceration, first of the kidney or its pelvis, or the top of the ureter, causes at the same time suppurative and adhesive inflammation, proceeding gradually to the surface, where it forms a prominent tumor, red, painful, soft, and fluctuating, and, either a spontaneous opening taking place or after an incision, matter is discharged, and not unusually with that one or more urinary calculi, or scabulous matter and urine. The swelling subsides after the first discharge of matter; but the aperture evinces no disposition to close, and matter continues to be discharged for months, or years, while a long sinus or fistula leading to the kidney is maintained. It is then a renal fistula, discharging matter, and sometimes urine and sand, or urinary concretions. If the opening happen to become closed, much pain is produced, and all the former symp-

2*

toms of nephritis ensue, until fresh suppuration takes place, and the aperture is reopened.*

This is a very good description of the case the particulars of which have been related in the preceding pages. In most of these cases, Dr. Cragie remarks, "the local disorder of the kidney, if it do not prove immediately fatal, gives rise to more or less hectic fever, with wasting and loss of strength." The favorable issue of our case was due to the fortunate escape of the calculus, which, by its presence, had excited and kept up the renal irritation. The quantity of pus discharged by the aperture grew less and less every day after the passage of the calculus, until it ceased altogether, and the orifice was permanently closed. There is reason to believe that the secretion of the diseased kidney continued all the time, for the amount of urine voided was about as large as natural; and it is certain that this fluid did not constitute any part of the contents of the tumor, for the pus never emitted at any time an ammoniacal or urinous odor.

June 30th, 1853.

* Elements of General aud Pathological Anatomy. By David Craigie, p. 941.

